# Biochemical and synergistic properties of a novel alpha‐amylase from Chinese nong‐flavor Daqu

**DOI:** 10.1186/s12934-021-01571-w

**Published:** 2021-04-07

**Authors:** Lanchai Chen, Zhuolin Yi, Yang Fang, Yanling Jin, Kaize He, Yao Xiao, Dong Zhao, Huibo Luo, Hui He, Qun Sun, Hai Zhao

**Affiliations:** 1 CAS Key Laboratory of Environmental and Applied Microbiology, Environmental Microbiology Key Laboratory of Sichuan Province, Chengdu Institute of Biology, Chinese Academy of Sciences, No. 9 Section 4, Renmin Nan Road, Chengdu, 610041 Sichuan People’s Republic of China; 2 Key Laboratory of Bio-Resource and Eco-Environment of Ministry of Education, College of Life Sciences, Sichuan University, No. 24 South Section 1, Yihuan Road, Chengdu, 610065 People’s Republic of China; 3 Analytical and Testing Center, Sichuan University of Science and Engineering, Zigong, 643000 China; 4 Wuliangye Group, Yibin, 644007 China; 5 Liquor Making Bio-Technology and Application of Key Laboratory of Sichuan Province, Bioengineering College, Sichuan University of Science and Engineering, Zigong, 643000 China; 6 Department of Liquor Making Engineering, Moutai College, Renhuai, 564501 China

**Keywords:** Alkali‐resisting α-amylase, GH13_5 subfamily, Synergistic action, Daqu

## Abstract

**Background:**

Daqu is the most important fermentation starter for Chinese liquor, with large number of microbes and enzymes being openly enriched in the Daqu system over thousands of years. However, only a few enzymes have been analyzed with crude protein for total liquefying power and saccharifying power of Daqu. Therefore, the complex enzymatic system present in Daqu has not been completely characterized. Moreover, their pivotal and complicated functions in Daqu are completely unknown.

**Results:**

In this study, a novel α-amylase NFAmy13B, from GH13_5 subfamily (according to the Carbohydrate-Active enZYmes Database, CAZy) was successfully heterologous expressed by *Escherichia coli* from Chinese Nong-flavor (NF) Daqu. It exhibited high stability ranging from pH 5.5 to 12.5, and higher specific activity, compared to other GH13_5 fungal α-amylases. Moreover, NFAmy13B did not show activity loss and retained 96% residual activity after pre-incubation at pH 11 for 21 h and pH 12 for 10 h, respectively. Additionally, 1.25 mM Ca^2+^ significantly improved its thermostability. NFAmy13B showed a synergistic effect on degrading wheat starch with NFAmy13A (GH13_1), another α-amylase from Daqu. Both enzymes could cleave maltotetraose and maltopentaose in same degradation pattern, and only NFAmy13A could efficiently degrade maltotriose. Moreover, NFAmy13B showed higher catalytic efficiency on long-chain starch, while NFAmy13A had higher catalytic efficiency on short-chain maltooligosaccharides. Their different catalytic efficiencies on starch and maltooligosaccharides may be caused by their discrepant substrate-binding region.

**Conclusions:**

This study mined a novel GH13_5 fungal α-amylase (NFAmy13B) with outstanding alkali resistance from Nong-flavor (NF) Daqu. Furthermore, its synergistic effect with NFAmy13A (GH13_1) on hydrolyzing wheat starch was confirmed, and their possible contribution in NF Daqu was also speculated. Thus, we not only provide a candidate α-amylase for industry, but also a useful strategy for further studying the interactions in the complex enzyme system of Daqu.

**Supplementary Information:**

The online version contains supplementary material available at 10.1186/s12934-021-01571-w.

## Background

Normally, to efficiently degrade polysaccharides in nature, microbes produce a set of enzymes with collaborative activities, such as endoglucanase, cellobiohydrolase, and β-glycosidase on cellulose [1], xylanase and β-xylosidase on xylan [2], and α-amylase, β-amylase, and glucoamylase on starch [3]. Even enzymes with the same degradation pattern may exhibit synergistic action, for example, the endoglucanases of Cel5 and Cel9 from *Paenibacillus panacisoli* [4], the xylanases of XT6 from *Geobacillus stearothermophilus*, Xyn2A from *Trichoderma viride* [5], and the α-amylases of ApAmy59 and ApAmy80 from *Aplysia kurodai* [6]. Among carbohydrate degrading enzymes, α-amylase (EC 3.2.1.1) is an endo-acting amylolytic enzyme, cleaving the internal α-1,4-glucosidic bonds in starch, and has been widely used in various fields, such as the food, detergent, pharmaceutical, and textile industries. Glycoside Hydrolases Family GH13 is the main representative of α-amylases and has been divided into several subfamilies (e.g. GH13_1, 5, 6, 7, 15, 24, 27, 28, 32, 36, and 37) based on the correlation between sequence and enzymatic specificity [7, 8]. A number of α-amylases have been identified from individual microbes, such as eight α-amylases from *Aspergillus niger* [9] and two α-amylase isoforms from *Aspergillus oryzae* [10]. However, most of these α-amylases have only been investigated for their primary features, and their potential competition and synergistic effects have seldom been compared.

Nong flavor (NF) Daqu is the most important fermentation starter for Chinese liquor, with a large number of microbes and enzymes being openly enriched in the Daqu system over thousands of years [11]. However, only a few enzymes have been obtained using culture-dependent methods, such as thermostable α-amylase from *Bacillus* in Daqu [12], with most being analyzed with crude protein for total liquefying and saccharifying power [13]. Therefore, the complex enzymatic system present in Daqu has not been completely characterized. Until recently, a great breakthrough was made in the metatranscriptomic analysis of NF Daqu, with a total of 932 carbohydrate-active enzymes identified in the high-temperature stage (N3) [14]. Subsequently, a fungal α-amylase, NFAmy13A [15], and an endoglucanase, NFEg16A [16], were mined and characterized from NF Daqu.

In addition, amylases are the main contributor to the liquefying and saccharifying processes of Daqu, which have a significant effect on the quality of Daqu [13]. Based on previous metatranscriptomic analysis, α-amylase exhibited the second highest expression level with 465.7 RPKM (reads per kilobase per million) among enzymes related to starch metabolism [14]. Hence, α-amylase may play an important role in the NF Daqu system. Based on our unpublished data, a total of 15 α-amylases were successfully identified at the N3 stage of NF Daqu, among which 10 belonged to GH13_1 and 5 belonged to GH13_5. The vast majority of fungal α-amylases have been assigned to the GH13_1 and 5 subfamilies. GH13_1 is composed of extracellular α-amylases originating from fungi and yeast, where the representative enzyme is Taka-amylase from *Aspergillus oryzae* [17]. On the other hand, intracellular fungal α-amylases are classified into the GH13_5 subfamily, which includes AmyD from *Aspergillus niger* [18]. However, until now, no studies have reported on the interaction between these two subfamily members or their contributions in natural environment. Thus, NF Daqu is a good resource for studying the competition and collaboration between α-amylases.

A novel fungal α-amylase (NFAmy13B), recognized as a GH13_5 member, was successfully obtained from the high-temperature stage of NF Daqu and characterized in this study. The first α-amylase previously characterized, NFAmy13A [15], was assigned to GH13_1. Thus, the gene product belonging to the GH13_5 subfamily was chosen here to investigate the possible synergistic action between α-amylases from the GH13_1 and 5 subfamilies. Herein, we provide an efficient strategy to mine novel enzymes, shedding light on their pivotal role in the fermentation of Daqu and possibly improving the production and quality of this Chinese liquor in the future.

## Materials and methods

### Materials

Mix (Green) for PCR amplification and pDE2 Directional TOPO Expression Kit for expression vector construction were purchased from TsingKe (Beijing, China). The Universal DNA Purification Kit used for PCR product purification was purchased from TianGen (Beijing, China). *Escherichia coli* Trans5ɑ and BL21(DE3) were purchased from TransGen Biotech (Beijing, China) and used for gene cloning and protein expression, respectively. Sonics Vibra-Cell™ used for cell disruption was purchased from Sonics & Materials (Newtown, USA). Ni-NTA Sefinose™ Resin was obtained from Sangon Biotech (Shanghai, China) for protein purification. Amicon® Ultra-15 Centrifugal Filter Devices (Merck Millipore, Germany) were used for protein concentration. NanoDrop 2000c (Thermo Fisher Scientific, Waltham, MA) was used to analyze the concentrations of DNA and protein. High-performance anion-exchange chromatography (HPAEC) (Dionex ICS-5000) with a pulsed amperometric detector (PAD) was obtained from Thermo Fisher Scientific (Waltham, MA). Kanamycin sulphate, isopropylthio-β-galactoside (IPTG), imidazole, and other chemical reagents used in this study were purchased from yuanye Bio-Technology (Shanghai, China).

### Gene cloning and expression vector construction

The gene product of ORF 22,984 was the second α-amylase characterized in the GH13 family in the NF Daqu system, and was named NFAmy13B. Similar to previous studies [15], the gene fragments were directly amplified by Mix (Green) with N3 cDNA as its template and two primers (NFABf (CACCATGAAGTCCCTCCTCTGCTGC) and NFABr (CTAGTGCTTGTAGATATCCGAGTC)). The gene fragments were analyzed on 1% agarose gel and purified using a Universal DNA Purification Kit. The purified DNA fragments were ligated into the pDE2 vector according to the manufacturer’s instructions. Trans5α was used as the host strain for the transformation of recombinant pDE2 with the *NFAmy13B* gene. Transformants were selected on Luria-Bertani (LB) medium containing kanamycin (50 µg/mL). Positive clones with the correct inserts were verified by DNA sequencing (Sangon Biotech).

### Gene expression and protein purification

The recombinant pDE2-*NFAmy13B* plasmid was transformed into BL21 (DE3) for gene expression. After an overnight culture on LB plate supplemented with the same antibiotic, a single colony was picked and inoculated into 3 mL LB at 37 °C with shaking (200 rpm) for incubation overnight. Subsequently, 1 mL of pre-culture was inoculated into 1 L of LB medium containing the same antibiotic as described above at 37 °C and 200 rpm. When OD_600_ reached 0.5–0.8, 0.1 mM IPTG was added to the culture to induce gene expression, and the temperature was decreased to 16 °C. After 24 h of cultivation, the cells were harvested by centrifugation at 8000 rpm and 4 °C for 3 min, then suspended in binding buffer (50 mM Tris-HCl, 300 mM NaCl, pH 7.5). The cells were disrupted by sonication, and the ruptured cells were clarified by centrifugation at 8000 rpm for 10 min at 4 °C to remove cell debris. The supernatant was then applied to Ni-NTA agarose resin for further purification, according to the manufacturer’s instruction with some modifications. The loaded protein was washed with binding buffer supplemented with 20 mM imidazole until only small amounts of protein were eluted. The bound protein was eluted using binding buffer supplemented with 250 mM imidazole, and fractions were collected for further analysis. The purity and molecular mass of the protein were determined by sodium dodecyl sulfate-polyacrylamide gel electrophoresis (SDS-PAGE). According to the results of SDS-PAGE, the fractions with the desired proteins were collected and concentrated in a protein storage buffer (50 mM Tris-HCl, 150 mM NaCl, pH 7.5) using Amicon® Ultra-15 centrifugal filters. The concentration of the purified protein was analyzed using NanoDrop 2000c.

### Plate‐based activity assay of NFAmy13B

To investigate the potential activity of NFAmy13B, a plate-based activity assay was performed. Solid plates were prepared by dissolving 0.1% AZCL-dextran, -arabinoxylan, -HE-cellulose, -pullulan, -amylose, -xylan, or -debranched arabinan (Megazyme) in phosphate buffer at pH 6.0 supplemented with 1% agarose. Approximate 20 µg of purified protein was added to the center hole of the plates. The plates were subsequently incubated at 54 °C overnight, and amylase activity was determined by the blue zone around the hole of the plates.

### Thermal and pH stability assays

The NFAmy13B activity was performed at 54 °C and pH 6.0 for 30 min throughout this work, and the assay without enzyme was used as a blank control. The amount of reducing ends released was detected with glucose as the standard using the para-hydroxybenzoic acid hydrazide (*p*HBAH; Sigma-Aldrich, St. Louis, MO) method [19].

To evaluate the pH stability of NFAmy13B, 600 nM purified enzyme was incubated in various pHs, ranging from 3.0 to 13.0 (50 mM citrate buffer for pH 3.0–6.0, 50 mM phosphate buffer for pH 6.0–8.0, 50 mM Tris-HCl buffer for pH 8.0–9.5, 25 mM Na_2_CO_3–_NaOH buffer for pH 9.5–1.0, 25 mM Na_2_HPO_4–_NaOH buffer for 11.0–12.0, and 50 mM KCl–NaOH buffer for 12.0–13.0) at 20 °C for 1 h. Furthermore, to evaluate the alkali-resisting characteristic of NFAmy13B, 600 nM enzyme was pre-treated at pH 11.0, 12.0, and 13.0 for different times, respectively. Then, the residual activity was measured by incubating 40 nM enzyme and 2 mg/mL corn starch at 54 °C and pH 6.0 for 30 min. The residual activity was calculated by assuming the untreated control as 100%.

To determine its thermostability at different temperatures, 150 nM enzyme was first pre-treated in 50 mM phosphate buffer (pH 6.0) at 40 to 64 °C for 30 min on a 96-well thermal cycler (Bio-Rad, San Diego, CA, USA). After cooling on ice, the residual activities were determined at 54 °C and pH 6.0 for 30 min. To detect the half-life of thermal inactivation at 45, 50, 55, and 60 °C, 75 nM enzyme was pre-treated at the stated temperatures for different periods of time with or without 1.25 mM CaCl_2_. Their residual activities were determined with 30 nM enzyme and 5 mg/mL substrate at 54 °C and pH 6.0 for 30 min. The enzyme activity of the untreated control was designated as 100%. Its thermal inactivation half-life (t_1/2_) was obtained by plotting the natural logarithms of the residual activity values versus incubation time and calculated from linear regression.

### Effects of chemical compounds on enzyme activity

The effect of various additives (1 and 10 mM, except 1 and 10% for ethanol) on NFAmy13B activity was investigated by incubating the enzyme with corn starch in the presence of different chemical compounds at 54 °C for 30 min. The released reducing ends were determined using the *p*HBAH assay, and the relative activities were expressed as a percentage of the activity of the unadded control, designated as 100%.

### Substrate specificity and steady‐state kinetic analysis of NFAmy13B

Firstly, the digesting capacities of NFAmy13B on various substrates were determined by incubating 40 nM enzyme with 5 mg/mL substrates (e.g., soluble starch, amylopectin, amylose, wheat starch, potato starch, corn starch, xylan, laminarin, dextrin, glycogen, pullulan, and arabinoxylan) at 54 °C for 30 min. The released amount of reducing sugars was estimated using the *p*HBAH method. Secondly, its specific activities were determined by incubating a low concentration of 20 nM enzyme with various substrates (5 mg/mL) containing 1.25 mM CaCl_2_ for different times, where the slopes of the reducing products against time were linear. One unit of enzyme activity was defined as the amount of enzyme required to release 1 µmol glucose equivalents (reducing ends) from a specific substrate per minute under standard conditions. Lastly, to investigate the kinetic parameters, 20 nM enzyme was incubated with starch substrates (corn starch, potato starch, wheat starch, amylose, and amylopectin) ranging from 0.1 to 16 mg/mL at 54 °C for 20 min. The velocities were kept in a constant scope under every condition by using low concentration of 20 nM enzyme. The initial velocities were plotted against the substrate concentrations, and their kinetic values were determined by applying the Michaelis-Menten equation using a nonlinear regression method (Origin 9).

### Time‐course hydrolysis of different concentrations of starchy substrates

Initially, the experiments were performed by incubating a constant concentration of 8 mg/mL starchy substrates (potato starch, corn starch, and wheat starch) with a high concentration of 1 µM protein in the presence of 1.25 mM CaCl_2_ at 54 °C and pH 6.0 for different time intervals (2, 4, 8, 12, 16, and 20 h). The released reducing ends (glucose equivalents) were quantified using the *p*HBAH method. Meanwhile, a constant incubation time of 12 h and various concentrations (1 to 16 mg/mL) of starchy substrates were studied under the same conditions. The end-products were analyzed by HPAEC-PAD using a Dionex CarboPac PA20 analytical column (3 × 150 mm) and Dionex CarboPac PA20 guard column (3 × 30 mm). The mobile phase comprised component A (250 mM NaOH) and B (10 mM NaOH, 500 mM NaOAc). Glucose (M1), maltose (M2), maltotriose (M3), maltotetraose (M4), and amylopentaose (M5) were used as standards.

### Hydrolysis of NFAmy13B and NFAmy13A on maltooligosaccharides and wheat starch

To effectively evaluate the synergistic hydrolysis of NFAmy13B and NFAmy13A on wheat starch, different concentrations of NFAmy13B and NFAmy13A were used to release the same reducing ends from wheat starch. Thus, group of 20 nM NFAmy13B and 1000 nM NFAmy13A were set with equivalent relative activity. Different ratios of 20 nM NFAmy13B and 1000 nM NFAmy13A were combined to get the equivalent relative activity with each of 20 nM NFAmy13B and 1000 nM NFAmy13A. Therefore, their synergistic hydrolysis was carried out by incubating different ratios of 20 nM NFAmy13B and 1000 nM NFAmy13A with 5 mg/mL wheat starch in the presence of 1.25 mM CaCl_2_ at 54 °C for 30 min. The soluble end-products were analyzed by HPACE-PAD. To confirm the hydrolysis patterns of these two enzymes, the hydrolysis of maltooligosaccharides was studied at 54 °C and pH 6.0 for various time periods (5 min, 20 min, 60 min and 2 h) by incubating 1 µM NFAmy13B or NFAmy13A with 5 mM maltose (M2), maltotriose (M3), maltotetraose (M4), and amylopentaose (M5), respectively, and analyzed using the HPAEC-PAD method.

### Phylogenetic analysis of NFAmy13B and NFAmy13A

The amino acid sequences used for phylogenetic tree construction in the phylogenetic analysis are listed in Additional file 1: Table S1. The α-amylase alignments were conducted using the MUSCLE method [20], and their phylogenetic relationships were analyzed using Mega 7 software. The statistical method was neighbor-joining, and the bootstrap replications were 1000.

### Protein structure modeling

The three-dimensional (3D) structure of NFAmy13A and NFAmy13B was constructed by homology-based modeling on SWISS-MODEL (https://swissmodel.expasy.org/) using PDB 3KWX and 1WPC as a template, respectively. All of the predicted structures were visualized by PyMOL software version 2.3.3 (PyMOL Molecular Graphics System, Schrödinger, LLC).

### Nucleotide sequence accession number

The nucleotide sequence of *NFAmy13B* obtained from the N3 cDNA library was deposited in the NCBI GenBank database under Accession Number MT849765.

## Results

### Cloning, expression, and purification of amylase

**Fig. 1:**
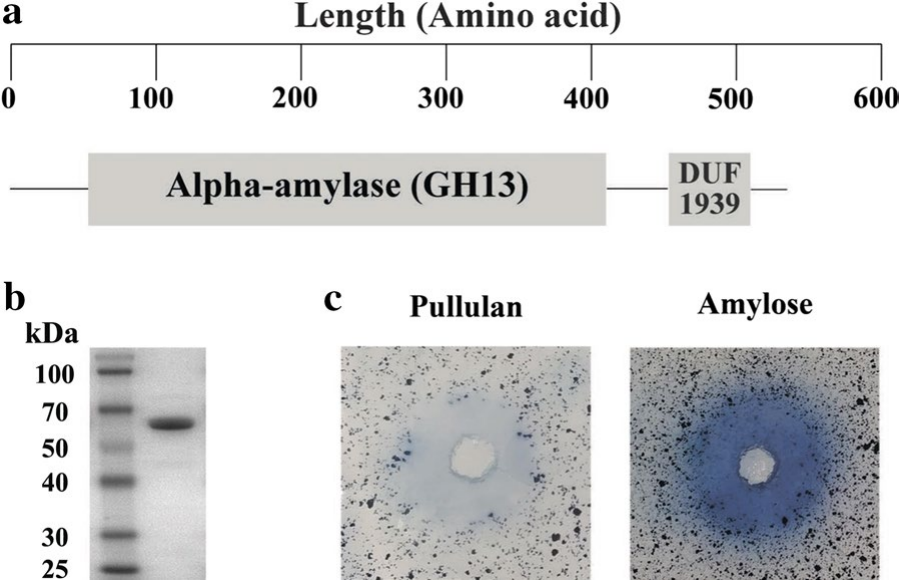
Schematic representation, purification and plate-based activity assays of NFAmy13A. Scheme (**a**), SDS-PAGE analysis (**b**), and plate-based activity assay (**c**) of NFAmy13B. GH13: family 13 glycoside hydrolase domain. DUF1939: domain of unknown function 1939

The gene sequence of *NFAmy13B* was successfully obtained by specific amplification with primers NFABf/r from N3-cDNA and expressed in *E. coli* BL21(DE3). The recombinant NFAmy13B with a C-terminal 6×His tag was purified by affinity chromatography using Ni^2+^-NTA resin. Based on our previous metatranscriptomic analysis of Daqu N3 [14], ORF 22,984 was predicted to encode an enzyme with an alpha-amylase domain and DUF1939 (domain of unknown function) (Fig. 1a). The molecular mass of the purified NFAmy13B corresponded well with its predicted value (61.05 k) (Fig. 1b). NFAmy13B showed clear activity in degrading amylose and pullulan (Fig. 1c).

### pH and thermal stability of NFAmy13B

**Fig. 2:**
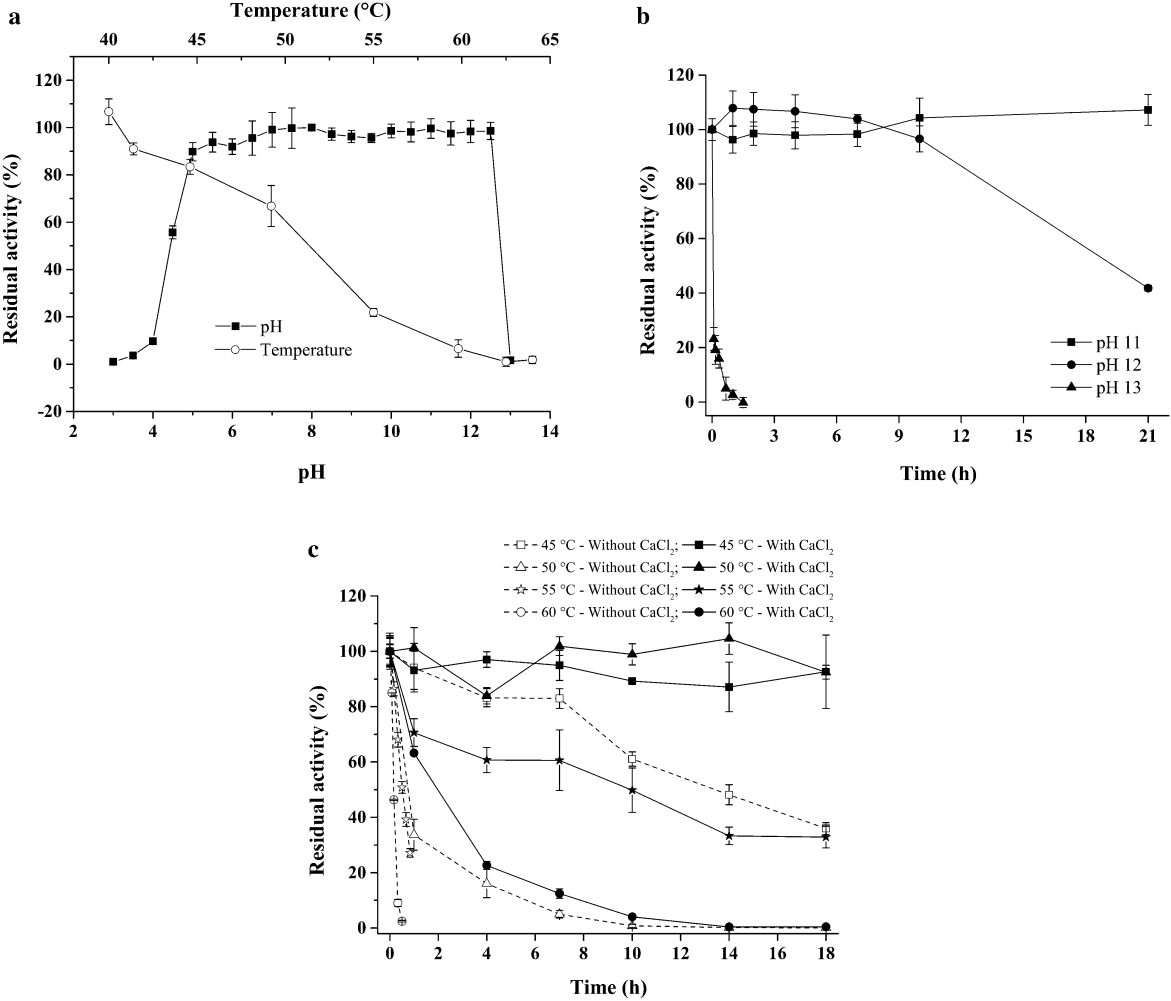
Effects of pH and temperature on the stability of NFAmy13B. The pH and temperature stability were determined from pH 3 to 13 and from 40 to 64 °C, respectively (**a**); Stability of NFAmy13B at pH 11, 12, and 13 were detected with different incubation times (**b**); Thermostability of NFAmy13B at 45, 50, 55, and 60 °C were measured with or without CaCl_2_, respectively (**c**). Each experiment was performed in quadruplicate

After pre-treatment at various pH values at 20 °C for 1 h, NFAmy13B was found to be very stable at a pH range of 5.5–12.5, with over 90% residual activity (Fig. 2a). Moreover, NFAmy13B was stable at pH 11 and pH 12, with almost no activity loss after pre-incubation at pH 11 for 21 h and 96% residual activity after pre-treatment at pH 12 for 10 h (Fig. 2b). After pre-incubation at 40 to 64 °C for 30 min, the residual activity of NFAmy13B decreased rapidly with an increasing temperature, and retained over 60% residual activity between 40 and 50 °C (Fig. 2a).

To evaluate the effect of divalent calcium ions on the thermostability of NFAmy13B, different concentrations (0 ~ 25 mM) of Ca^2+^ were pre-incubated with the enzyme at 55 °C for 30 min, and its reducing ends released was subsequently measured. In the presence of Ca^2+^, the enzyme released higher amount of reducing ends at 0.0025–6.25 mM than enzymes without Ca^2+^ (Additional file 1: Fig. S1). The highest amount of reducing ends was observed at 1.25 mM Ca^2+^, clearly indicating the ability of Ca^2+^ to stimulate the thermostability of NFAmy13B (Additional file 1: Fig. S1). In the presence and absence of 1.25 mM Ca^2+^, the thermostability of NFAmy13B was further studied at 45, 50, 55, and 60 °C. After incubating at 45 and 50 °C for 18 h, NFAmy13B lost almost 64 and 100% activity, respectively, while in the presence of Ca^2+^ enzyme retained around 90% activity during the same pretreatment (Fig. 2c). Moreover, after pre-incubation at high temperatures (55 and 60 °C) for 1 h, NFAmy13B quickly lost its activity and retained < 27% residual activity, while the presence of Ca^2+^ largely increased its residual activity to 74% at 55 °C and 63% at 60 °C (Fig. 2c). In other words, in the presence of 1.25 mM Ca^2+^, the thermal half-lives of NFAmy13B at 55 and 60 °C were enhanced from 0.5 to 0.1 h to 9 and 2 h, respectively.

### Effect of chemical reagents on the activity of purified NFAmy13B

The effects of additives on enzymatic activity were determined with final concentrations of 1 mM and 10 mM (Table 1). The activity of NFAmy13B was not influenced by 1 mM Ca^2+^; slightly inhibited by K^+^ at both concentrations, and Na^+^ only at 1 mM; moderately inhibited by Ba^2+^ and Li^+^ at 1 mM; largely inhibited by the remaining metal ions. None of the detected additives showed any stimulatory effect on its activity.

**Table 1:** Effect of chemicals on the activity of NFAmy13B

Additives	Relative activity (%) (mean ± standard deviation)	
	1 mM	10 mM
Control	100 ± 2	100 ± 2
CaCl_2_	95.9 ± 4.4	42.7 ± 3.1***
NiCl_2_	44.9 ± 1.8***	22.8 ± 7.2***
NH_4_Cl	88.7 ± 1.2***	78.6 ± 0.5***
BaCl_2_	90.7 ± 6.7**	39.7 ± 2.6***
KCl	92.8 ± 4.0*	89.0 ± 7.2*
Ethanol^a^	79.0 ± 3.6***	59.4 ± 8.3***
CoCl_2_·6H_2_O	71.0 ± 4.0***	0***
FeCl_3_	16.5 ± 3.5***	0.576 ± 1.724***
MnCl_2_·4H_2_O	61.5 ± 3.0***	0***
ZnSO_4_	5.78 ± 0.17***	1.36 ± 0.50***
CuSO_4_	4.69 ± 0.10***	1.10 ± 3.68***
EDTA·Na_2_	66.0 ± 0.8***	31.2 ± 1.4***
HgCl_2_	8.15 ± 0.47***	2.53 ± 0.59***
AlCl_3_	15.6 ± 0.9***	0.889 ± 0.637***
MgCl_2_	78.3 ± 2.8***	78.9 ± 2.9***
SDS	3.22 ± 0.13***	2.02 ± 0.39***
LiCl	90.5 ± 6.3**	86.9 ± 0.4***
NaCl	92.3 ± 0.6*	82.1 ± 0.1***

^a^The concentrations of ethanol were 1 and 10%

*0.01 < p < 0.5; **: 0.001 < p < 0.01; ***: p < 0.001. Each experiment was performed in triplicate

### Substrate specificity and kinetic parameters of NFAmy13B

NFAmy13B efficiently degraded various starchy substrates and showed the highest activity toward amylopectin (Additional file 1: Table S2). Some degrading capacities were also observed for dextrin, glycogen, and pullulan, while no activity was found for xylan, laminarin, arabinoxylan, and cellulose. NFAmy13B showed the highest specific activity of 151.9 U/mg on potato starch, and had higher specific activities on amylopectin (151.7 U/mg) and corn starch (143.3 U/mg) than on wheat starch (74.0 U/mg) and amylose (73.4 U/mg).

The kinetic hydrolysis of starchy substrates by NFAmy13B did not fit very well to the Michaelis-Menten equation, especially potato starch which was fail to regression (Additional file 1: Fig. S2). Among detected substrates, NFAmy13B definitely showed the lowest velocity on amylose at > 3 mg/mL concentrations.

### Time course hydrolysis of various concentrations of starchy substrates

**Fig. 3:**
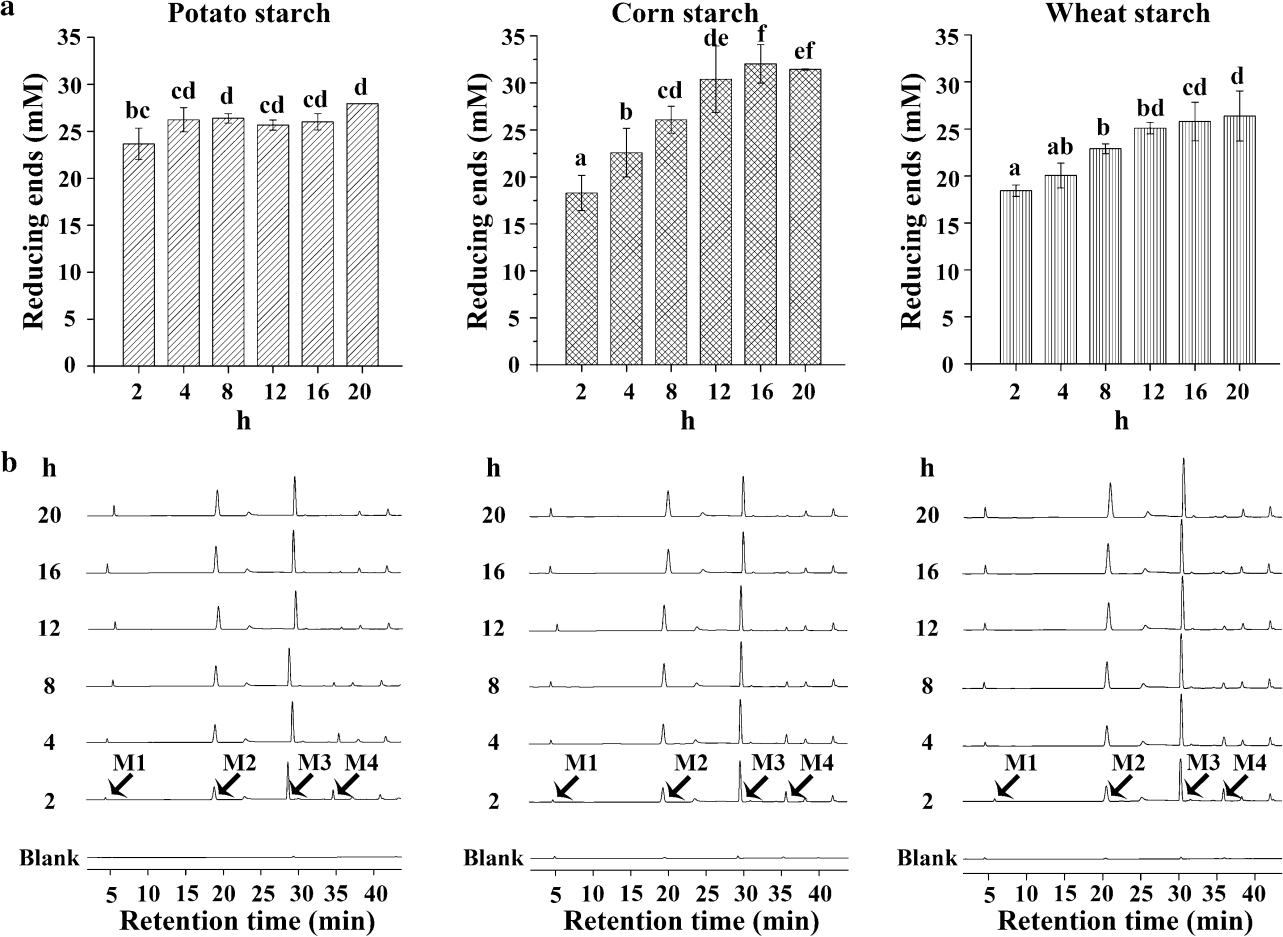
Hydrolysis of starchy substrates for various times by NFAmy13B. Eight mg/mL potato, corn, and wheat starch were hydrolyzed by NFAmy13B for 2 to 20 h in the presence of 1.25 mM CaCl_2_, and the products were hydrolyzed by reducing ends (**a**) and HPAEC-PAD (**b**). M1, glucose; M2, maltose; M3, maltotriose; M4, maltotetraose. Note: Means with different small letters are significantly different (p < 0.05). Each experiment was performed in triplicate

A high concentration of 1 µM NFAmy13B was first applied to hydrolyze 8 mg/mL potato starch, corn starch, and wheat starch for different times. As shown in Fig. 3a, a large increase of the reducing products was quickly released from potato starch at early time points of 2 h and products were almost unchanged in the subsequent hours. On the other hand, for corn starch and wheat starch, the released reducing products showed clear increases upon incubation, and almost reached constant values after 16 and 12 h, respectively. Among the three starchy substrates, in the short time of 2 h, more products were released from potato starch, and in the long incubating period of 16 h, more were released from corn starch. The main end-products were maltose (M2) and maltotriose (M3) for the three substrates, while small amounts of glucose (M1) and maltotetraose (M4) were observed throughout the incubation period (Fig. 3b). M3 was the highest proportion of end-products, with the amount of M1–M3 increasing and M4 decreasing during incubation.

**Fig. 4:**
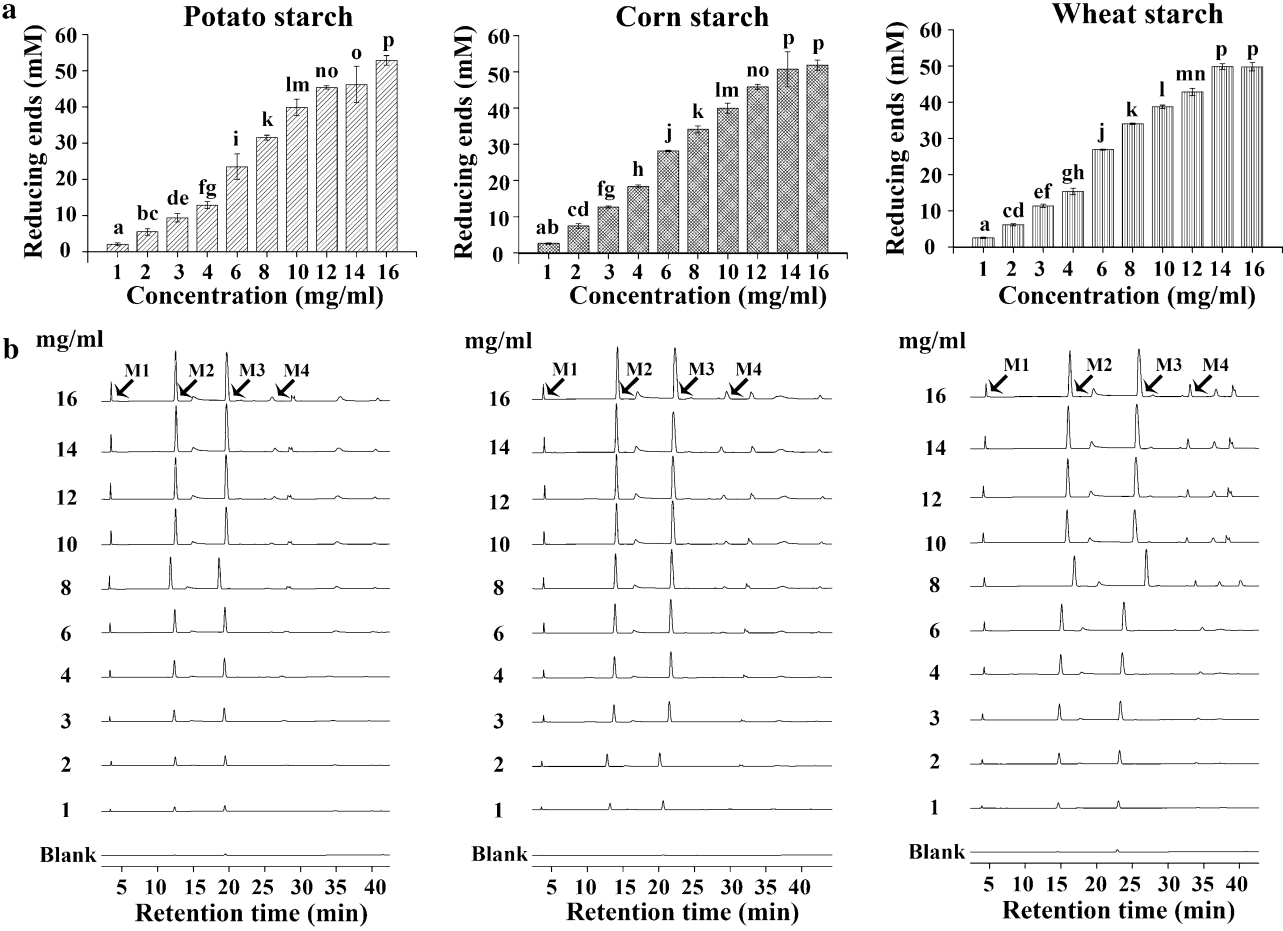
Hydrolysis of increasing amount of starchy substrates by NFAmy13B. Substrates with different concentrations (1 to 16 mg/mL) of potato, corn, and wheat starch were hydrolyzed by NFAmy13B in the presence of 1.25 mM CaCl_2_, and the products were hydrolyzed by reducing ends (**a**) and HPAEC-PAD (**b**). M1, glucose; M2, maltose; M3, maltotriose; M4, maltotetraose. Note: Means with different small letters are significantly different (p < 0.05). Each experiment was performed in triplicate

Secondly, a high concentration of 1 µM NFAmy13B was used to hydrolyze different concentrations of the three starchy substrates for a constant incubation time of 12 h. As shown in Fig. 4a, the amounts of released reducing ends increased with the concentrations of the three substrates, reaching the highest points at 16 mg/mL from potato starch, ~ 14 mg/mL from corn starch and wheat starch. The main end-products of M2 and M3 and small amounts of M1 were detected at all concentrations, while minor amounts of M4 were only observed at higher concentrations (Fig. 4b). All end-products increased with the concentrations of the three substrates.

### Synergistic actions between NFAmy13A and NFAmy13B

Since NFAmy13A and NFAmy13B were both obtained from Chinese NF Daqu, their potential synergistic actions were studied here. Under the same conditions (54 °C and pH 6.0), NFAmy13A showed a lower specific activity for wheat starch (4.85 U/mg) than NFAmy13B (74.0 U/mg), indicating that NFAmy13A had a lower degrading capacity for the long-chain starchy substrates. Therefore, different concentrations of NFAmy13B (20 nM) and NFAmy13A (1000 nM) were set to release the same reducing ends from wheat starch (Additional file 1: Table S3), and their potential synergistic effects were secondly studied with different enzyme ratios on wheat starch. As shown in Table 2, except the group of 12.5% NFAmy13B and 87.5% NFAmy13A, all combinations released more reducing ends than the individual. Moreover, the amount of M1 increased with the proportion of NFAmy13A, while M3 decreased. M4 was only detected in NFAmy13B alone. Therefore, these results indicate that NFAmy13B exerted a good synergistic effect with NFAmy13A for the degradation of starchy substrates.

**Table 2:** The synergistic activity between NFAmy13A and NFAmy13B on wheat starch

Various combinations		End-products (mM) (mean ± standard deviation)				Total sugars of M1 −M4 (mM) (mean ± standard deviation)	Total reducing ends^*^ (mM) (mean ± standard deviation)
NFAmy13B (20 nM)	NFAmy13A (1000 nM)	M1	M2	M3	M4		
0	100%	1.70 ± 0.11	5.78 ± 0.23	0.142 ± 0.024	0	7.62 ± 0.35^c^	9.77 ± 0.39^ cd^
12.5%	87.5%	1.83 ± 0.17	6.18 ± 0.43	0.281 ± 0.064	0	7.91 ± 0.08^bc^	9.97 ± 0.49^bcd^
25%	75%	1.73 ± 0.04	6.21 ± 0.16	0.445 ± 0.013	0	8.38 ± 0.21^a^	11.0 ± 0.5^a^
37.5%	62.5%	1.51 ± 0.04	5.95 ± 0.12	0.591 ± 0.025	0	8.05 ± 0.11^ab^	11.0 ± 0.3^a^
50%	50%	1.17 ± 0.07	5.66 ± 0.09	0.873 ± 0.050	0	7.70 ± 0.19^bc^	10.4 ± 0.3^abc^
62.5%	37.5%	0.889 ± 0.071	5.43 ± 0.23	1.09 ± 0.34	0	7.73 ± 0.19^bc^	11.1 ± 0.8^a^
75%	25%	0.390 ± 0.012	5.10 ± 0.14	1.74 ± 0.09	0	7.23 ± 0.23^d^	11.0 ± 0.4^a^
87.5%	12.5%	0	4.26 ± 0.11	2.37 ± 0.09	0	6.63 ± 0.18^e^	10.7 ± 0.4^ab^
100%	0	0	0.420 ± 0.139	3.22 ± 0.06	0.253 ± 0.024	3.89 ± 0.21^f^	9.36 ± 0.45^d^

* Total reducing ends were determined by the *p*HBAH method

Means with different superscript letters within a column are significantly different (p < 0.05). Each experiment was performed in triplicate

**Fig. 5:**
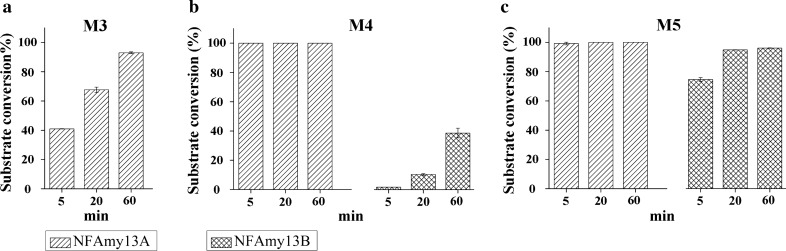
Substrate conversions of NFAmy13A and NFAmy13B on maltooligosaccharides. Five mM maltotriose (M3) (**a**), maltotetraose (M4) (**b**), and amylopentaose (M5) (**c**) were incubated with 1 µM NFAmy13B or NFAmy13A for various time periods (5 min, 20 min, 60 min and 2 h), respectively, and their conversions were calculated according to HPAEC-PAD analysis. Each experiment was performed in triplicate

Next, the hydrolysis patterns of NFAmy13A and NFAmy13B were identified for oligosaccharides. Firstly, 1 µM NFAmy13A or NFAmy13B was independently incubated with 5 mM M2 or M3 for 2 h. As shown in Additional file 1: Fig. S3, NFAmy13B showed no activity on M2 and M3. By contrast, NFAmy13A was unable to hydrolyze M2, but completely hydrolyzed M3 into M1 and M2. To compare their catalytic efficiency on maltooligosaccharides in detail, short incubation periods of 5 min, 20 and 60 min were performed to hydrolyze M3, M4, and M5. As shown in Fig. 5 and Additional file 1: Table S4, for each substrate, the catalytic efficiency of NFAmy13A was higher than that of NFAmy13B. The hydrolyzation of M3 by NFAmy13A increased with the incubation periods, and M3 was completely hydrolyzed into M1 and M2 after 1 h. NFAmy13A degraded M4 into M1, M2, and M3 after a short incubating period of 5 min, and completely hydrolyzed M4 into M1 and M2 in 1 h. Meanwhile, NFAmy13B partly converted M4 into major M2, with minor amounts of M1 and M3 during the different incubation periods, and a substrate conversion of only 62.5% after incubation for 1 h. Moreover, NFAmy13A completely degraded M5 into M1, M2, and M3 after 5 min, and its major end-products were M1 and M2 after 1 h. NFAmy13B also efficiently degraded M5 into M2 and M3, with trace amounts of M1, and its substrate conversion increased from 71.5% to 5 min to 99.2% after 1 h. These results indicate that same hydrolyzing patterns were identified between NFAmy13A and NFAmy13B on maltooligosaccharides except M3, and NFAmy13A exhibited a higher catalytic efficiency than NFAmy13B.

### Bioinformatics analysis of NFAmy13B and NFAmy13A

The amino acid sequence of NFAmy13B showed the highest similarity (77.6%) to the putative α-amylase (XP_028489551.1) from *Byssochlamys spectabilis*. Meanwhile, NFAmy13B shared a high sequence similarity (62.7%) with the characterized intracellular fungal α-amylase (AmyD) from *Aspergillus niger* [9, 18], and 47.5% with crystallized maltohexaose-producing amylase (G6-amylase, PDB: 1WP6) from alkalophilic *Bacillus* sp.707 [21]. Based on the phylogenetic analysis (Additional file 1: Fig. S4), NFAmy13A was categorized into subfamily GH13_1, which is designated as eukaryotic extracellular α-amylases from fungi, while NFAmy13B was categorized as GH 13_5, which includes bacterial liquefying α-amylases, intracellular fungal α-amylases, and some archaea α-amylases [7]. AmyD was representative of fungal α-amylase in the GH13_5 subfamily, and shared several conserved amino acids, which were all conserved in NFAmy13B (Additional file 1: Fig. S5). NFAmy13B had no signal sequence predicted by SignalP-5.0 Server (http://www.cbs.dtu.dk/services/SignalP/) (Additional file 1: Fig. S4). Meanwhile, compared with bacterial G6-amylase, three catalytic residues (Asp236, Glu266, and Asp333) and nine substrate binding sites (‒6 to + 3) in 1WPC were also highly conserved in NFAmy13B (Additional file 1: Fig. S5), indicating similar degrading patterns between them. Furthermore, four out of seven metal ion binding sites were also conserved in NFAmy13B (Additional file 1: Fig. S5).

**Fig. 6:**
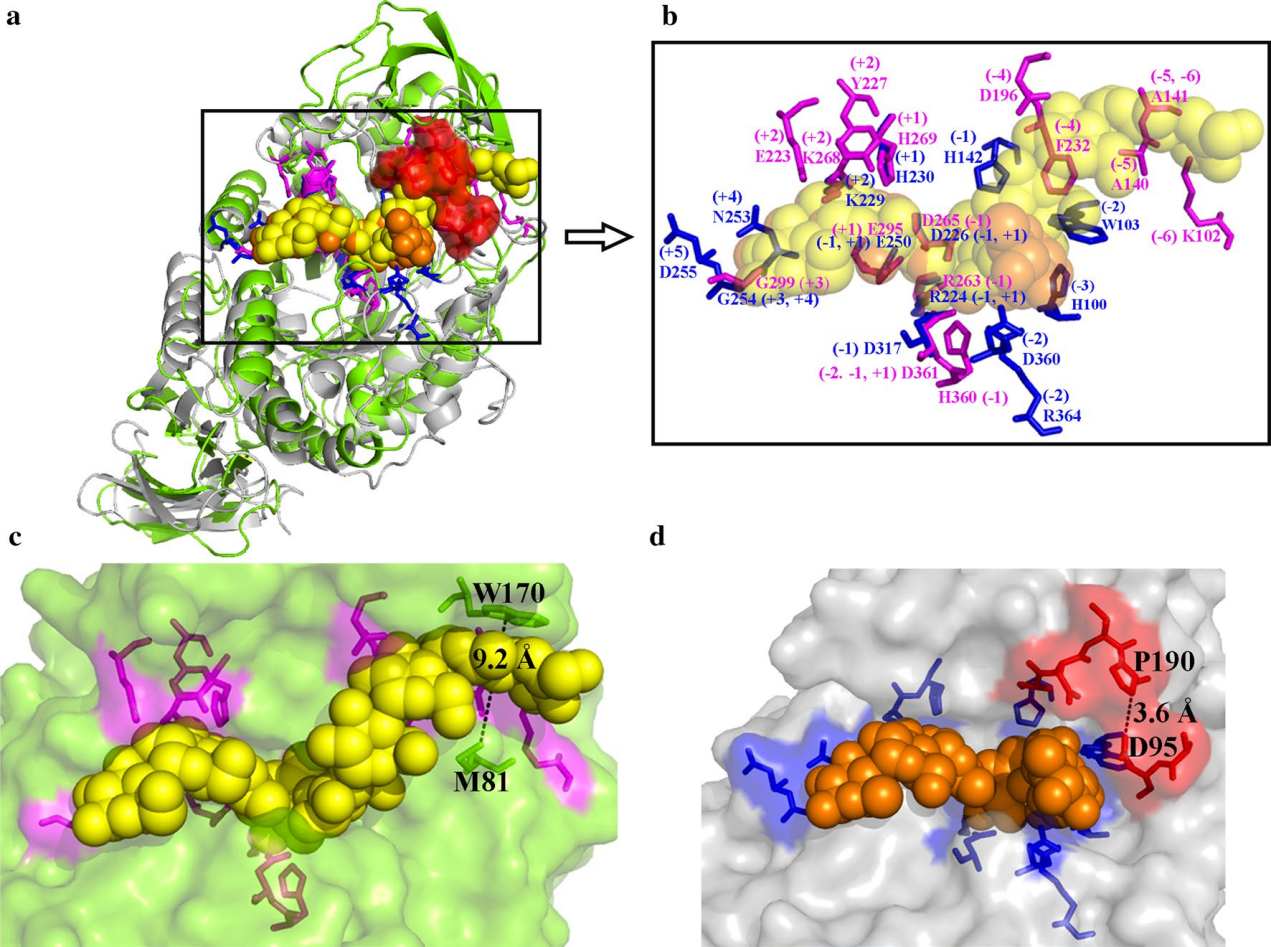
Three-dimensional structure alignment of NFAmy13B and NFAmy13A. The overall structure of NFAmy13B (green) and NFAmy13A (gray) were constructed using PDB 1WPC and 3KWX, and showed in cartoon style (**a**); The substrate-binding sites of NFAmy13B and NFAmy13A are shown as magenta and blue sticks, respectively (**b**); The surface presentation showed the shape of the pocket, and yellow and orange spheres represent the substrates binding with NFAmy13B (**c**) and NFAmy13A (**d**), respectively. Red sticks and surface area indicate the block region of NFAmy13A compared with NFAmy13B

NFAmy13B showed a low identity of 13.3% with NFAmy13A (Additional file 1: Fig. S5), and their 3D structures were conservatively folded into the N-terminal catalytic (β/α)_8_-barrel domain and C-terminal eight-stranded β-sandwich domain (Fig. 6a). In detail, NFAmy13B has 9 potential conserved substrate-binding sites (as described above), while NFAmy13A may have 8 potential conserved substrate-binding sites (‒3 to + 5) (Fig. 6b, Additional file 1: Fig. S5). Thus, completely conserved substrate binding sites were observed between NFAmy13B and NFAmy13A at ‒1 to + 1 and + 3, and partly conserved at + 2, whereas large differences were observed at other binding sites. As shown in Fig. 6c, a wide active cleft was identified around the ‒5 and ‒6 sites of NFAmy13B, with the closest distance of 9.2 Å between W170 and M81, while the active cleft in NFAmy13A was blocked by the ADTP (187 –190) and AD (94 –95) amino acid sequences, with a shorter distance of 3.6 Å between P190 and D95 (Additional file 1: Fig. S5, Fig. 6d).

## Discussion

Large amounts of microbes and enzymes have been openly and continually enriched in the Chinese Daqu system for centuries. Recently, a total of 932 carbohydrate-active enzymes were identified from the high-temperature stage (62 °C) of Chinese NF Daqu by metatranscriptomics [14]. To mine these functional enzymes from the NF Daqu system, a sequence-based metatranscriptomic method was established in our previous study [15]. Compared with the random screening of function-based metatranscriptomic methods, this method involved rational and efficient screening allowing to directly obtain enzymes. Based on this method, α-amylase NFAmy13A [15] and endoglucanase NFEg16A [16] were successfully mined from NF Daqu, both of which showed high expression levels at high-temperature stage. Besides the highly expressed enzymes, some low expressed enzymes were also identified with whole length gene sequences, and were found to play complementary roles in Daqu system, which could also be easily and efficiently mined using this sequence-based metatranscriptomic method. Therefore, in the present study, a novel α-amylase (NFAmy13B) with low level of expression (RPKM < 0.02) was successfully obtained from the high-temperature stage of NF Daqu, and its potential roles in Daqu and industrial applications were speculated.

According to the sequence alignment, the *NFAmy13B* gene may originate from a fungal genus, such as *Byssochlamys*. Besides, NFAmy13B may be an intracellular fungal α-amylase based on the phylogenetic analysis. Interestingly, NFAmy13B was very stable at a pH range of 5.5–12.5 (Fig. 2a), with no activity loss at pH 11 for 21 h and keeping 96% residual activity at pH 12 for 10 h (Fig. 2b) This was more stable than most fungal alkali-resisting amylases (Additional file 1: Table S5) [22, 23] and was markedly different from intracellular fungal α-amylase AmyD (stable at pH 4–6) and AmyF (stable at pH 3–5) [9]. Moreover, compared to most bacteria alkali-resisting amylases, NFAmy13B still showed a better stability at these pH values (Additional file 1: Table S5), except α-amylase from *Bacillus* sp., which showed a higher stability at pH 13 and a lower stability at pH 6–12 [24]. As a consequence, NFAmy13B, a fungal α-amylase, showed an extraordinary alkali-resisting behavior at pH 5.5–12.5 among these reports, indicating its potential application in the alkaline process of cotton desizing in the textile and detergent industries [25].

Meanwhile, NFAmy13B, a mesophilic α-amylase, was unstable at > 50 °C, with thermal half-lives of 0.8 h at 50 °C, 0.5 h at 55 °C, and 0.1 h at 60 °C (Fig. 2c). In the presence of 1.25 mM Ca^2+^, its thermal half-lives were markedly improved, rising to > 18 h, 9 h, and 2 h, respectively. This appears to be consistent with its partly conserved Ca^2+^ binding sites (Additional file 1: Fig. S5). However, the intracellular fungal α-amylases, AmyD and AmyF, both showed maximal activity at 30 °C and were only stable below 40 °C [9]. The thermostabilities of some amylases were also promoted by an appropriate concentration of Ca^2+^ [26–28], since the combination of calcium with their binding sites could improve the structural integrity [29, 30] and reduce flexibility [31, 32]. However, no further stimulation of the activity was observed for NFAmy13B by Ca^2+^ (Table 1). Similar results were also observed for α-amylase from thermophilic *Geobacillus thermoleovorans*, and its thermostability, not activity, was found to be similarly improved by Ca^2+^ [33]. On the other hand, the activity and thermostability of certain α-amylases were stimulated by Ca^2+^ [15, 27], while the rest were Ca^2+^ independent, without any stimulation of their activity and thermostability [18, 34–36].

For other chemical reagents, none showed any stimulation of the activity for NFAmy13B, indicating that it was a metal ion-independent enzyme. In contrast, most of chemicals significantly inhibited its activity at concentrations of 1 mM and 10 mM, while Co^2+^ and Mn^2+^ completely inhibited its activity at 10 mM (Table 1). Among these inhibitors, Hg^2+^ was commonly observed to strongly inhibit α-amylases, which might result from changes in the overall structure and competitive binding to the active site [37]. Fe^3+^, Zn^2+^, Cu^2+^, and Al^3+^ also exhibited similar levels of inhibition of α-amylase from *Nesterenkonia* sp. [38, 39], whereas Fe^3+^ and Cu^2+^ were found to stimulate α-amylase AmyP activity in a marine metagenomic library [40]. In some cases, Co^2+^ and Mn^2+^ could be used as accelerants to promote the activities of α-amylases [40–42].

As shown in Additional file 1: Table S2, NFAmy13B was able to hydrolyze a broad range of starchy substrates, showing the highest degradation capacities for amylopectin and potato starch, followed by corn starch, wheat starch, and amylose, in accordance with α-amylase from *Geomyces pannorum* and NFAmy13A from NF Daqu [15, 43]. Its specific activity of 151.9 U/mg on potato starch was much higher than 2.2 U/mg of the representative intracellular fungal α-amylase AmyD [18]. Amylopectin is constituted of α-(1–4)-linked D-glucosyl units in chains interconnected by α-(1–6)-linked D-glucosidic linkages, more end chains of which would be attacked than that of amylose, and the higher proportion of amylopectin in starchy substrates resulting in easier degradation [44]. Moreover, the preferences of NFAmy13B and NFAmy13A for amylopectin over amylose would confirm their key roles in the fermentation of NF baijiu, since the wheat material of NF Daqu and the sorghum material of alcohol fermentation both have higher amylopectin contents [45, 46]. In addition, to mimic the starchy degradation in the NF Daqu system, high concentration of NFAmy13B and starchy substrates were incubated together for different times. As a result, NFAmy13B was found to efficiently release more reducing ends from potato starch, corn starch, and wheat starch upon increasing periods, up to ~ 4 h, ~ 16 h, and ~ 12 h, respectively (Fig. 3a), and showed a good tolerance to high concentrations of 16 mg/mL for potato starch, ~ 14 mg/mL for corn starch and wheat starch (Fig. 4a). The hydrolysis products were mainly maltose and maltotriose with a small quantity of glucose (Figs. 3b and 4b), which was similar to AmyD and AmyF from *Aspergillus niger* [9].

In our previous study, NFAmy13A was the first α-amylase to be directly mined from Chinese NF Daqu. Its main products were maltose, with a small amount of glucose [15] which was markedly different from NFAmy13B. Meanwhile, NFAmy13A showed high expression level with an RPKM value of 293.5 at the high-temperature stage of NF Daqu, while NFAmy13B showed low expression level with a RPKM of < 0.02. Therefore, NFAmy13A and NFAmy13B may play different contribution on the process of making Daqu. Their complementary actions to hydrolyze wheat starch may verify our hypothesis in some degree.

**Fig. 7:**
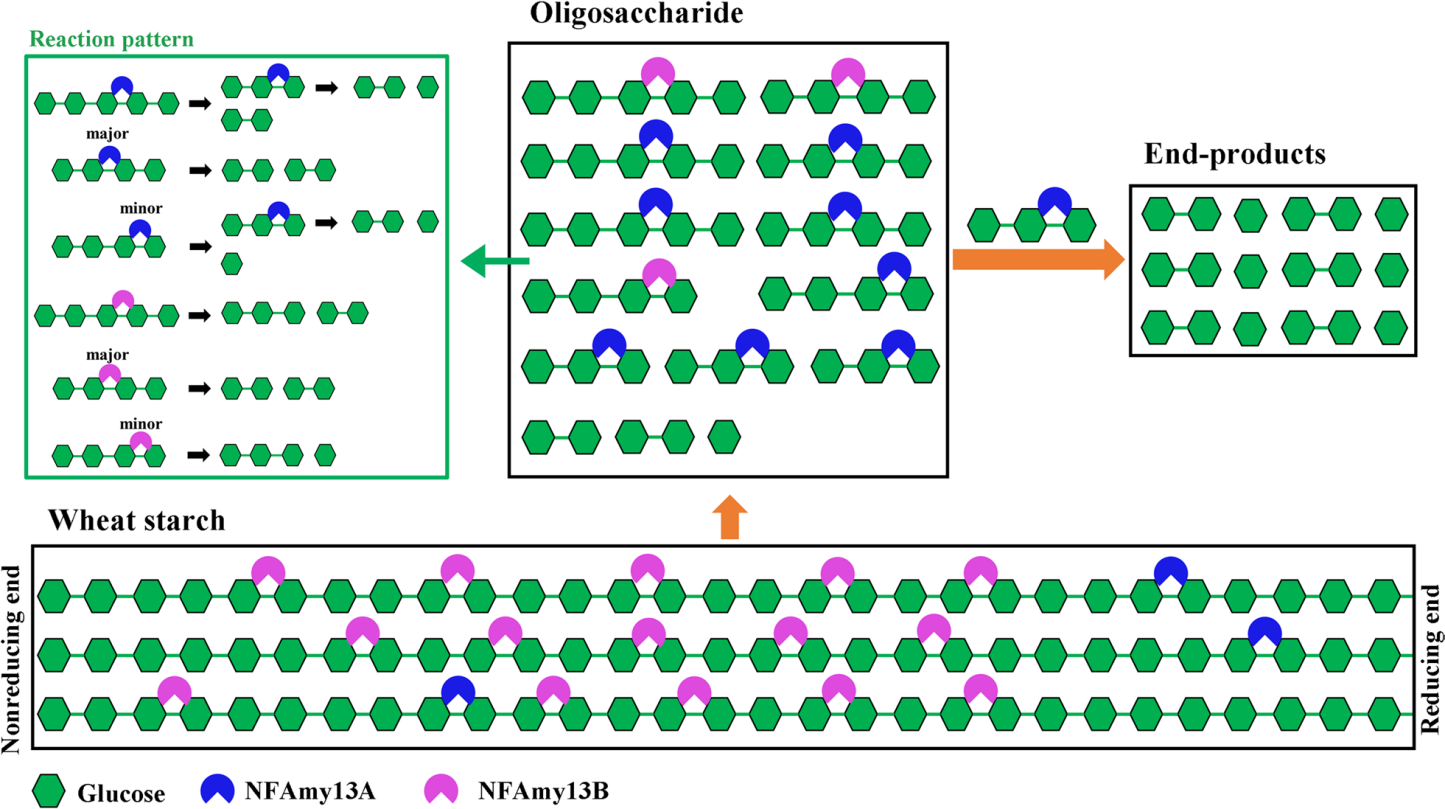
Starchy degradation schematic of NFAmy13A and NFAmy13B. The orange bold arrows indicated the process of synergistical degradation of starch and maltooligosaccharides by NFAmy13A and NFAmy13B. The green thin arrow showed the reaction patterns of NFAmy13A and NFAmy13B toward maltooligosaccharides

The hydrolyzing patterns were further confirmed between NFAmy13B and NFAmy13A on maltooligosaccharides (Fig. 5, Additional file 1: Table S4). NFAmy13B showed no hydrolytic capacity toward maltose and maltotriose, which was in accordance with α-amylases from *Clostridia* [47], *Lipomyces starkeyi* [48], and *Brachybacterium* sp. [49]. On the other hand, NFAmy13A was unable to hydrolyze maltose, but hydrolyzed maltotriose to glucose and maltose, which was similarly observed for α-amylases from *Paenibacillus* sp. [50], *Thermotoga neapolitana* [51], and *Rhizopus oryzae* [52]. Since maltose showed the highest proportion (59%) in the end-products of NFAmy13A (Table 2), NFAmy13A may also be clarified as a maltogenic α-amylase. Moreover, according to the degradation of M4 and M5, same reaction patterns could be speculated between NFAmy13A and NFAmy13B, namely, both cleaved M5 into M2 and M3, and mainly hydrolyzed M4 into M2 and M2, with a minor degradation of M4 into M1 and M3 (Addiitonal file 1: Table S4, Fig. 7). The same reaction patterns of M4 and M5 are partly consistent with their highly conserved substrate-binding sites (‒1 to + 1 and + 3) (Fig. 6b, Additional file 1: Fig. S5). In short, NFAmy13B exhibited a similar reaction pattern, lower degrading capacities for short-chain maltooligosaccharide (M3, M4, and M5), and higher degrading capacities on long-chain starchy substrate, acting as a complement to NFAmy13A. These results are partly agreement with our finding that the longer active cleft of NFAmy13B than NFAmy13A (Fig. 6c, d). Therefore, it is reasonable to speculate that NFAmy13B could work efficiently as a liquefying α-amylase and played main contribution on hydrolyzing wheat starch to maltooligosaccharides in the liquefaction process, after which the maltooligosaccharide products were mainly hydrolyzed by maltogenic amylase NFAmy13A during saccharification process (Fig. 7). Further studies, including reverse transcription quantitative real-time PCR and metatranscriptomics, on more stages of the Daqu making process are needed to obtain more details on their efficient synergistic functions during NF Daqu fermentation. Considering their high synergistic effects on degrading starchy substrates and maltooligosaccharide products at high temperatures, NFAmy13B together with NFAmy13A are good candidates for improving the fermentation power of NF Daqu as well as in other traditional starch degradation industries.

## Conclusions

A novel α-amylase, designated as NFAmy13B, was successfully obtained from Daqu using metatranscriptomics and heterologous expression. Its biochemical properties were comprehensively investigated, and compared with other intracellular fungal α-amylases in the GH13_5 subfamily, NFAmy13B showed different properties in degrading starchy substrates, including a high stability over a wide pH range (5.5–12.5), a higher degrading capacity on potato starch, and higher levels of activity on amylopectin than on amylose. In addition to these characteristics, its thermostability, but not enzyme activity, was stimulated by Ca^2+^ and showed a tolerance to high substrate concentrations. More importantly, a high synergistic effect on degrading wheat starch was observed between NFAmy13B and NFAmy13A (GH13_1), another α-amylase with high level of expression at the same stage of NF Daqu. The degradation patterns on maltooligosaccharide except maltotriose were observed same between them. Meanwhile, NFAmy13B showed a higher catalytic efficiency on long-chain starches compared to short-chain maltooligosaccharides for NFAmy13A. Thus, we postulate that NFAmy13B plays the dominate role in hydrolyzing starch into maltooligosaccharides, similar to a liquefying α-amylase, subsequently NFAmy13A plays main contribution to the hydrolysis of maltooligosaccharides in NF Daqu. Therefore, our proposed strategy is an effective approach for directly mining novel enzymes from Daqu, and NFAmy13B together with NFAmy13A could be applied as candidate α-amylases in industries.

## Supplementary Information

**Additional file 1: Table S1.** Amino acid sequences used for phylogenetic tree construction. **Table S2** The hydrolysis of NFAmy13B on various substrates. **Table S3.** Degradation capacity of NFAmy13A and NFAmy13B on wheat starch. **Table S4.** The hydrolysis of NFAmy13A and NFAmy13B on maltooligosaccharides (M3−M5). **Table S5.** Comparison of alkali-resisting α-amylase. **Fig. S1.** Effect of the concentrations of calcium divalent ion on NFAmy13B thermostability. Enzyme (75 nM) was incubated with different concentrations of CaCl2 at pH 6.0 and 55 °C for 30 min. The residual activities were determined by incubating 30 nM enzyme with 5 mg/mL potato starch at 54 °C and pH 6.0 for 30 min. Each experiment was performed in triplicate. **Fig. S2.** Kinetic studies of the NFAmy13B on the different substrates. 20 nM enzyme with 1.25 mM CaCl2 was incubated with various concentrations substrate between 0.1 and 16 mg/mL at 54 °C for 20 min. Each experiment was performed in quadruplicate . **Fig. S3.** Hydrolysis of maltooligosaccharides by NFAmy13A and NFAmy13B. 5 mM M2 and M3 were hydrolyzed by 1 μM NFAmy13A or NFAmy13B in the presence of 1.25 mM CaCl2 at pH 6.0 and 54 °C for 2 h. The reaction products were analyzed by HPAEC-PAD. M1, glucose; M2, maltose; M3, maltotriose. **Fig. S4.** Phylogenetic analysisof NFAmy13A and NFAmy13B. The amino acid sequences of enzymes from GH13 used for the construction of the phylogenetic treewere selected according to the similarities with NFAmy13A or NFAmy13B. Each enzyme was indicateda numbercorresponding to theGH13 subfamily, its abbreviation, and source. Detailsareprovided in Table S1. **Fig. S5.** Amino acid sequences alignment in NFAmy13A/B and high-similarity α-amylases.

## Data Availability

All the data generated or analyzed during this study have been included in this published article.
