# Supplementary material for: Biochemical and synergistic properties of a novel alpha‐amylase from Chinese nong‐flavor Daqu
Source: Microb Cell Fact. 2021 Apr 7;20:80. doi: 10.1186/s12934-021-01571-w (PMC8028695; doi:10.1186/s12934-021-01571-w)
Supplement: Supplementary file 1 — Additional file 1: Table S1. Amino acid sequences used for phylogenetic tree construction. Table S2 The hydrolysis of NFAmy13B on various substrates. Table S3. Degradation capacity of NFAmy13A and NFAmy13B on wheat starch. Table S4. The hydrolysis of NFAmy13A and NFAmy13B on maltooligosaccharides (M3−M5). Table S5. Comparison of alkali-resisting α-amylase. Fig. S1. Effect of the concentrations of calcium divalent ion on NFAmy13B thermostability. Enzyme (75 nM) was incubated with different concentrations of CaCl2 at pH 6.0 and 55 °C for 30 min. The residual activities were determined by incubating 30 nM enzyme with 5 mg/mL potato starch at 54 °C and pH 6.0 for 30 min. Each experiment was performed in triplicate. Fig. S2. Kinetic studies of the NFAmy13B on the different substrates. 20 nM enzyme with 1.25 mM CaCl2 was incubated with various concentrations substrate between 0.1 and 16 mg/mL at 54 °C for 20 min. Each experiment was performed in quadruplicate . Fig. S3. Hydrolysis of maltooligosaccharides by NFAmy13A and NFAmy13B. 5 mM M2 and M3 were hydrolyzed by 1 μM NFAmy13A or NFAmy13B in the presence of 1.25 mM CaCl2 at pH 6.0 and 54 °C for 2 h. The reaction products were analyzed by HPAEC-PAD. M1, glucose; M2, maltose; M3, maltotriose. Fig. S4. Phylogenetic analysisof NFAmy13A and NFAmy13B. The amino acid sequences of enzymes from GH13 used for the construction of the phylogenetic treewere selected according to the similarities with NFAmy13A or NFAmy13B. Each enzyme was indicateda numbercorresponding to theGH13 subfamily, its abbreviation, and source. Detailsareprovided in Table S1. Fig. S5. Amino acid sequences alignment in NFAmy13A/B and high-similarity α-amylases. [file 12934_2021_1571_MOESM1_ESM.pdf]

Supplementary Material

Table S1. Amino acid sequences used for phylogenetic tree construction.

Source	Abbreviation	GH13 subfamily number	Sequence similarity[a] (%)	GenBank or PDB accession number
**Fungi (GH13_1)**				
Aspergillus oryzae	AmyA	1	64.4	2GUY_A
Malbranchea cinnamomea	McAmyA	1	59.0	3VM7_A
Aspergillus niger	α-amylase	1	55.3	2AAA_A
**Bacteria (GH13_2)**				
Bacillus stearothermophilus	Novamyl	2	26.9	1QHO_A
Thermoanaerobacterium thermosulfurigenes	CGTase	2	23.1	3BMV_A
**Fungi (GH13_5)**				
Paecilomyces variotii	alpha-amylase		77.6	XP_028489551.1
Aspergillus niger	AmyD	5	64.4	XP_001389762.2
Aspergillus niger	AmyF	5	54.5	XP_001393627.2
Paracoccidioides brasiliensis	Amy1	5	39.1	ABS11196.1
**Bacteria (GH13_5)**				
Bacillus sp. 707	G6-amylase	5	47.5	1WP6_A
Bacillus licheniformis	alpha-amylase	5	47.0	1OB0_A
Bacillus sp. Ksm-1378	AmyK	5	46.8	2DIE
Bacillus halmapalus	alpha-amylase	5	46.7	1W9X
Bacillus amyloliquefaciens	BAA	5	46.4	3BH4_A
Geobacillus stearothermophilus	AmyS	5	45.3	4UZU_A
Bacillus sp. strain KSM-K38	AmyK38	5	44.5	1UD2_A
Alicyclobacillus sp. 18711	AliC	5	44.2	6GXV_A
Halothermothrix orenii	AmyB	5	31.7	3BC9_A
**Plants (GH13_6)**				
Hordeum vulgare	Amy1	6	26.6	2QPS_A
Hordeum vulgare	Amy2	6	22.6	1AMY_A
**Archaea (GH13_7)**				
Pyrococcus woesei	PWA	7	45.0	1MWO_A
Pyrococcus woesei	alpha-amylase	7	28.2	3QGV_A
**Bacteria (GH13_10)**				
Deinococcus radiodurans R1	MTHase	10	33.8	2BHU_A
**Bacteria (GH13_20)**				
Thermus sp. IM6501	ThMA	20	24.7	1SMA_A
Geobacillus stearothermophilus	Neopullulanase	20	20.9	1J0H_A

**Archaea (GH13_20)**				
Thermococcus kodakarensis KOD1	TK-PUL	20	37.7	5OT1_A
**Bacteria (GH13_31)**				
Erwinia rhapontici	NX-5	31	24.5	4HOW_A
Listeria monocytogenes EGD-e	Lmo0184	31	23.9	5DO8_A
**Yeast (GH13_40)**				
Saccharomyces cerevisiae	Ima1	40	28.7	3AXH_A
**Bacteria (GH13_?)**				
Anoxybacillus ayderensis	ASKA	unknown	33.2	5A2A_A
Geobacillus thermoleovorans	GTA	unknown	32.2	4E2O_A

[a] The similarities of α-amylases were compared to NFAmy13B, except for those in group GH13_1, which were compared to NFAmy13A.

Table S2. The hydrolysis of NFAmy13B on various substrates.

Substrate	Main linkage/monomer	Glucose equivalents (mM) (mean ± standard deviation)
Soluble starch	α-(1→4)-α-(1→6) glucose	1.23±0.06
Amylopectin	α-(1→4)-α-(1→6) glucose	11.6±0.3
Amylose	α-(1→4) glucose	5.73±1.24
Wheat starch	α-(1→4)-α-(1→6) glucose	6.06±1.06
Potato starch	α-(1→4)-α-(1→6) glucose	5.42±0.71
Corn starch	α-(1→4)-α-(1→6) glucose	7.09±0.66
Xylan	β-(1→4) xylose	0
Laminarin	β-(1→3) and β-(1→6)-glucose	0
Dextrin	α-(1→4) and α-(1→4)-α-(1→6) glucose	6.79±0.85
Glycogen	α-(1→4)-α (1→6) glucose	0.225±0.030
Pullulan	α-(1→6) glucose	0.0692±0.0948
Arabinoxylan	β-(1→4) arabinose and xylose	0
Cellulose	β-(1→4) glucose	0

Each experiment was performed in triplicate.

Table S3. Degradation capacity of NFAmy13A and NFAmy13B on wheat starch.

Enzyme	Concentration (nM)	Reducing ends (mM)
NFAmy13B	20	6.71±0.46
NFAmy13A	900	4.67±0.30
	1000	6.63±1.16
	1100	7.70±1.75
	1200	7.18±1.62

Each experiment was performed in triplicate.

Table S4. The hydrolysis of NFAmy13A and NFAmy13B on maltooligosaccharides (M3~M5).

Substrate	Enzyme	Product component	The yield of reducing ends (mM) (mean ± standard deviation)		
			5 min	20 min	60 min
M3	NFAmy13A	M1	1.52±0.11	2.47±0.05	3.29±0.09
		M2	1.96±0.11	2.97±0.05	3.84±0.02
M4	NFAmy13A	M1	0.727±0.024	1.34±0.05	2.17±0.08
		M2	7.32±0.07	7.79±0.21	8.53±0.17
		M3	1.43±0.06	0.657±0.176	0
	NFAmy13B	M1	0.0984±0.0394	0.208±0.024	0.335±0.061
		M2	0.269±0.058	1.01±0.06	2.66±0.05
		M3	0.0867±0.1279	0.483±0.063	0.817±0.195
M5	NFAmy13A	M1	1.02±0.17	2.99±0.04	5.33±0.02
		M2	8.93±0.39	11.6±0.1	13.5±0.1
		M3	5.59±0.07	3.65±0.15	1.07±0.06
		M4	0	0	0
	NFAmy13B	M1	0	0.0951±0.770	0
		M2	5.81±0.03	6.94±0.07	7.18±0.04
		M3	7.05±0.22	8.18±0.09	7.99±0.21
		M4	0	0	0

Each experiment was performed in triplicate.

Table S5. Comparison of alkali-resisting α-amylases.

Amylase	Source	Optimum pH	pH stability[b]	References
NFAmy13B	Unknown	5.5~6.0	pH 5.5~12.5, 1 h, >90%	This study
G6-amylase	*Bacillus* sp.	8.8	pH 4.7~10.8, 30 min, >50%	[1]
*Td*AmyA	*Thermomyces dupontii*	6.5	pH 4.5~10.0, 30 min, >80%	[2]
Amy1	*Bacillus amyloliquefaciens*	6.0	pH 7.0~9.0, 1 h, 90%	[3]
α-amylase[a]	*Bacillus* sp.	5.0	pH 4.5~11.0, 1 h, 80%	[4]
α-amylase[a]	*Bacillus* sp.	10.5	pH 10.5, 15 h, 55%	[5]
α-amylase[a]	*Halorubrum xinjiangense*	8.5	pH 7~11, 1 h, >45%	[6]
α-amylase[a]	*Bacillus* sp.	8.0	pH 6~13, 30 min, >67%	[7]
TfAmy48	*Tepidimonas fonticaldi*	8.0	pH 11, 6 h, 50%	[8]
McAmyA[a]	*Malbranchea cinnamomea*	6.5	pH 5.0~10.0, 30 min, >90%	[9]

[a] Amylases were produced by the originated organism.
[b] This column indicates the residual activities of α-amylases pre-treated in the range of specified pH with designated time.

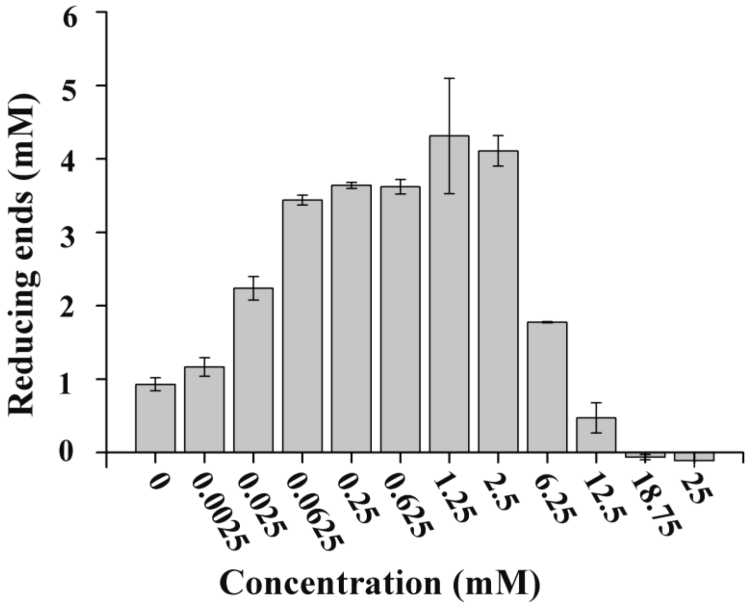

Fig. S1 Effect of the concentrations of calcium divalent ion on NFAmy13B thermostability. Enzyme (75 nM) was incubated with different concentrations of $CaCl_2$ at pH 6.0 and 55 °C for 30 min. The residual activities were determined by incubating 30 nM enzyme with 5 mg/mL potato starch at 54 °C and pH 6.0 for 30 min. Each experiment was performed in triplicate.

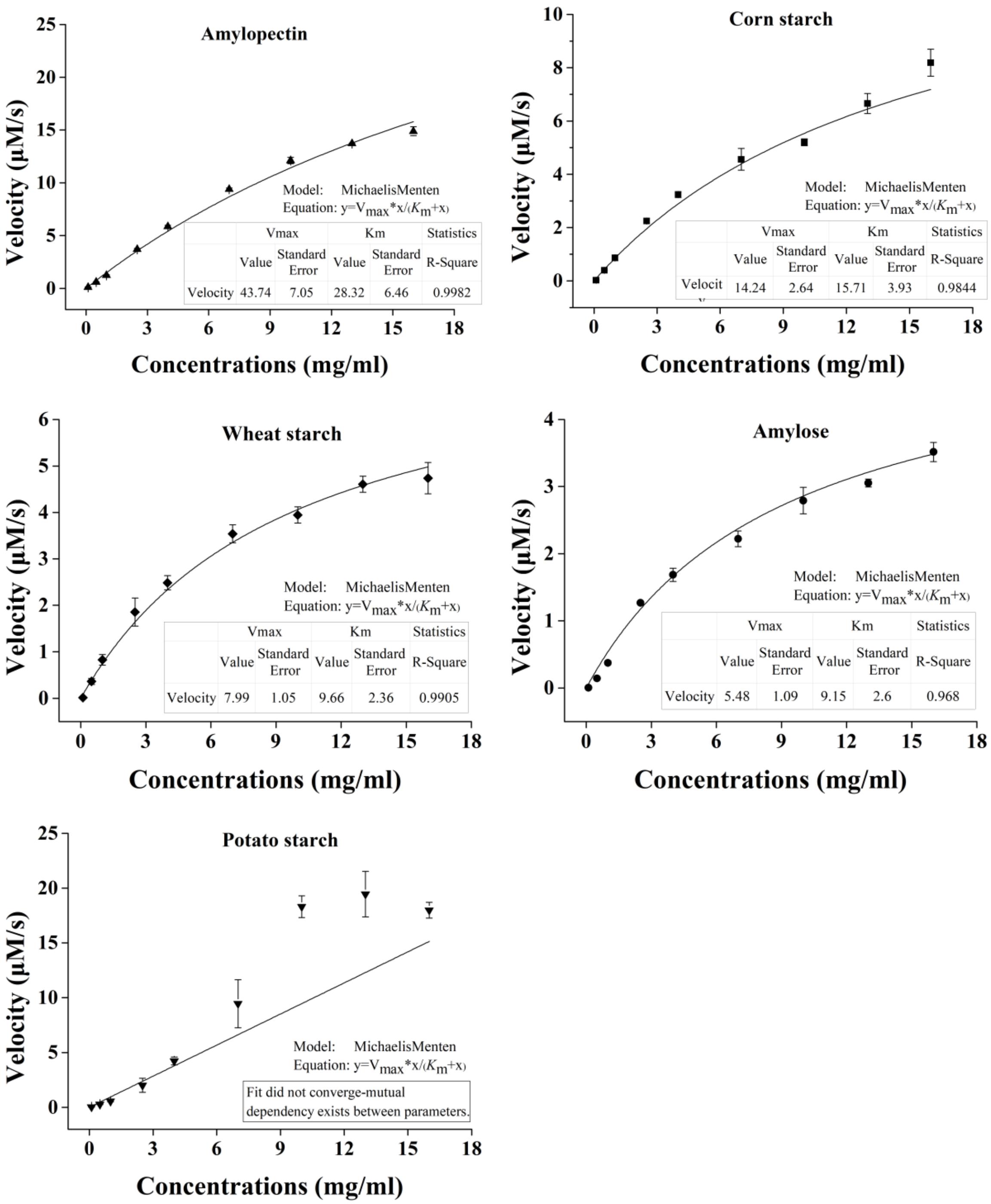

Fig. S2 Kinetic studies of the NFAmy13B on the different substrates. 20 nM enzyme with 1.25 mM $CaCl_2$ was incubated with various concentrations substrate between 0.1 and 16 mg/mL at 54 °C for 20 min. Each experiment was performed in quadruplicate.

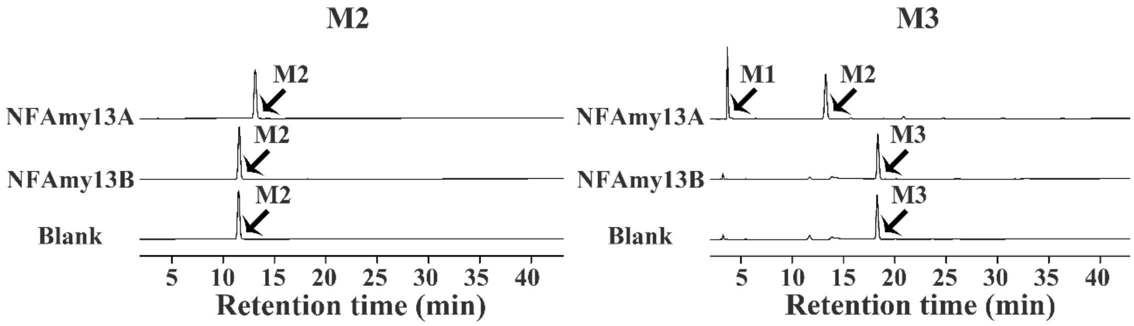

Fig. S3 Hydrolysis of maltooligosaccharides by NFAmy13A and NFAmy13B. 5 mM M2 and M3 were hydrolyzed by 1 μM NFAmy13A or NFAmy13B in the presence of 1.25 mM $CaCl_2$ at pH 6.0 and 54 °C for 2 h. The reaction products were analyzed by HPAEC-PAD. M1, glucose; M2, maltose; M3, maltotriose.

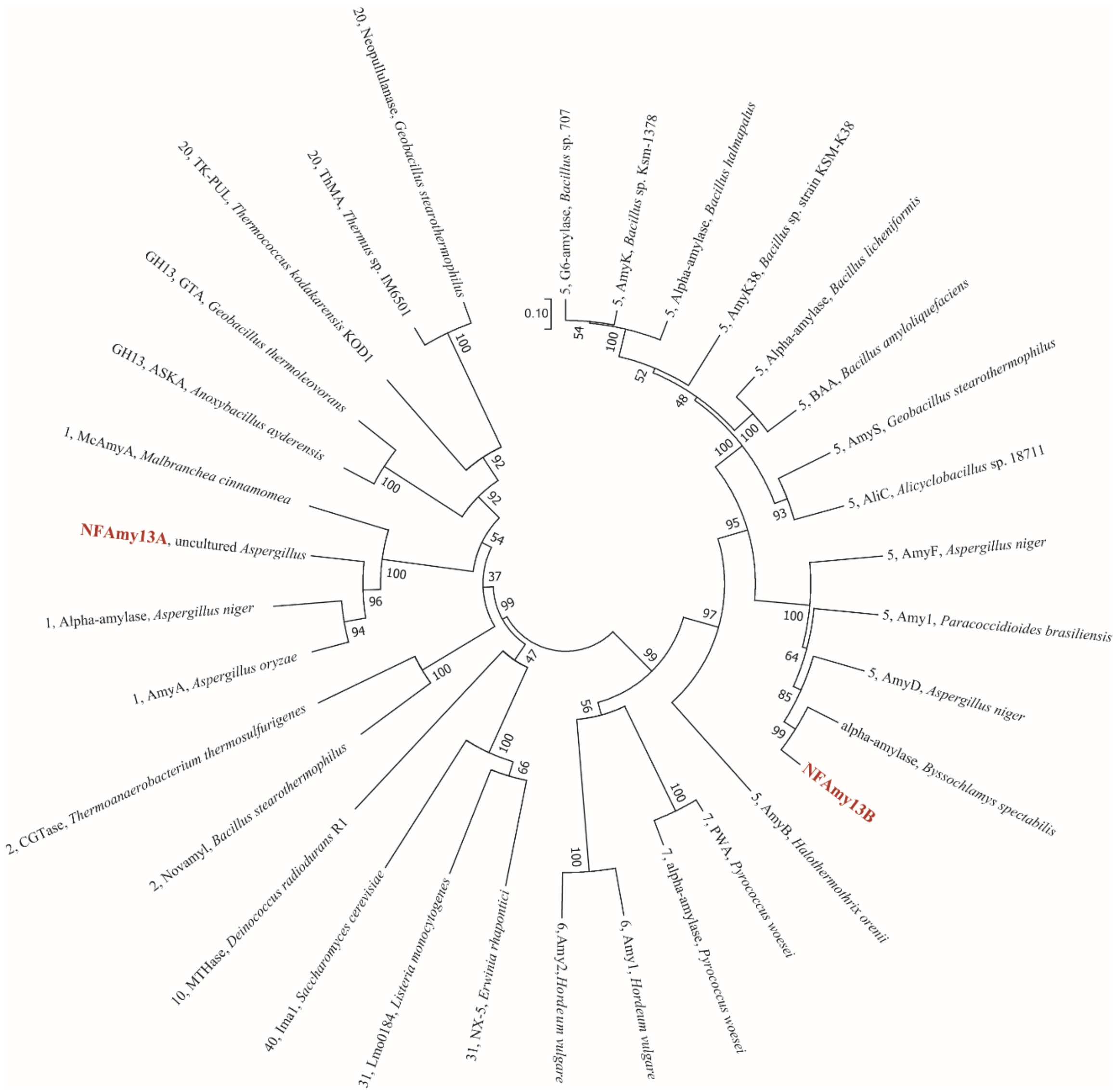

Fig. S4 Phylogenetic analysis of NFAmy13A and NFAmy13B. The amino acid sequences of enzymes from GH13 used for the construction of the phylogenetic tree were selected according to the similarities with NFAmy13A or NFAmy13B. Each enzyme was indicated a number corresponding to

the GH13 subfamily, its abbreviation, and source. Details are provided in Table S1.

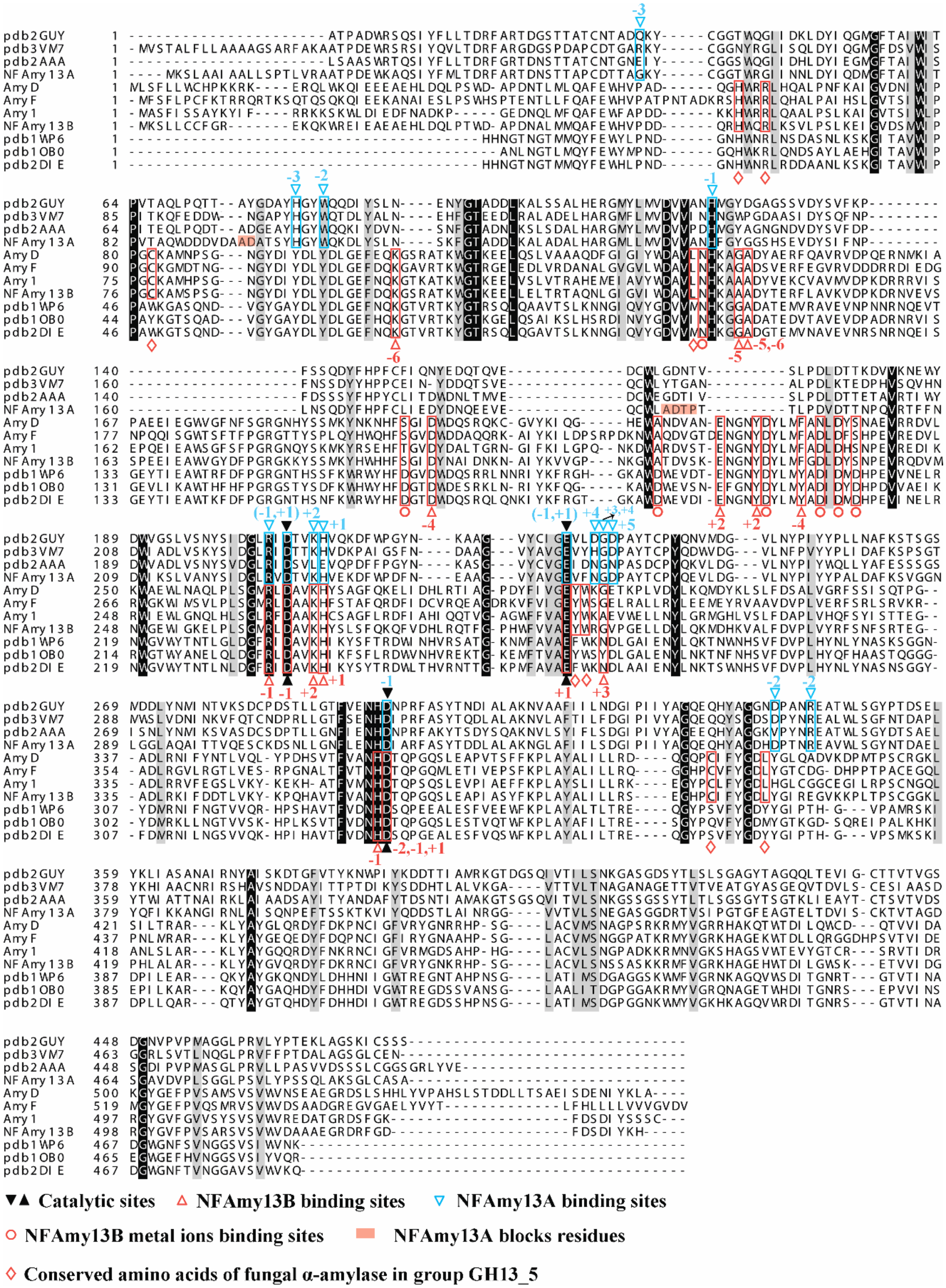

Fig. S5 Amino acid sequences alignment in NFAmy13A/B and high-similarity α-amylases.

References

1. Kanai R, Haga K, Akiba T, Yamane K, Harata K: **Biochemical and crystallographic analyses of maltohexaose-producing amylase from alkalophilic *Bacillus* sp. 707.** *Biochemistry* 2004, **43:**14047-14056.
2. Wang Y-c, Zhao N, Ma J-w, Liu J, Yan Q-j, Jiang Z-q: **High-level expression of a novel alpha-amylase from *Thermomyces dupontii* in *Pichia pastoris* and its application in maltose syrup production.** *Int J Biol Macromol* 2019, **127:**683-692.
3. Chen J, Chen X, Dai J, Xie G, Yan L, Lu L, Chen J: **Cloning, enhanced expression and characterization of an alpha-amylase gene from a wild strain in *B. subtilis* WB800.** *Int J Biol Macromol* 2015, **80:**200-207.
4. Liu XD, Xu Y: **A novel raw starch digesting alpha-amylase from a newly isolated *Bacillus* sp. YX-1: purification and characterization.** *Bioresour Technol* 2008, **99:**4315-4320.
5. Burhan A, Nisa U, Gökhan C, Ömer C, Ashabil A, Osman G: **Enzymatic properties of a novel thermostable, thermophilic, alkaline and chelator resistant amylase from an alkaliphilic *Bacillus* sp. isolate ANT-6.** *Process Biochem* 2003, **38:**1397-1403.
6. Moshfegh M, Shahverdi AR, Zarrini G, Faramarzi MA: **Biochemical characterization of an extracellular polyextremophilic alpha-amylase from the halophilic archaeon *Halorubrum xinjiangense*.** *Extremophiles* 2013, **17:**677-687.
7. Pancha I, Jain D, Shrivastav A, Mishra SK, Shethia B, Mishra S, Mohandas VP, Jha B: **A thermoactive alpha-amylase from a *Bacillus* sp. isolated from CSMCRI salt farm.** *Int J Biol Macromol* 2010, **47:**288-291.
8. Allala F, Bouacem K, Boucherba N, Azzouz Z, Mechri S, Sahnoun M, Benallaoua S, Hacene H, Jaouadi B, Bouanane-Darenfed A: **Purification, biochemical, and molecular characterization of a novel extracellular thermostable and alkaline alpha-amylase from *Tepidimonas fonticaldi* strain HB23.** *Int J Biol Macromol* 2019, **132:**558-574.
9. Han P, Zhou P, Hu S, Yang S, Yan Q, Jiang Z: **A novel multifunctional alpha-amylase from the thermophilic fungus *Malbranchea cinnamomea*: biochemical characterization and three-dimensional structure.** *Appl Biochem Biotechnol* 2013, **170:**420-435.
